# A word of caution: do not wake sleeping dogs; micrometastases of melanoma suddenly grew after progesterone treatment

**DOI:** 10.1186/1471-2407-13-132

**Published:** 2013-03-20

**Authors:** Jose Mordoh, Ivana Jaqueline Tapia, Maria Marcela Barrio

**Affiliations:** 1 Instituto Alexander Fleming, Cramer 1180, Buenos Aires, Argentina; 2 Centro de Investigaciones Oncológicas-Fundación Cáncer, Cramer 1180, Buenos Aires, Argentina

**Keywords:** Melanoma, Vaccine treatment, Progesterone treatment, Metastasis

## Abstract

**Background:**

Hormonal treatment might affect the immune response to tumor antigens induced in cancer patients who are being vaccinated.

**Case presentation:**

A 33 years-old woman was diagnosed with cutaneous melanoma in May 2009. Her melanoma was located in the intermammary sulcus, had a Breslow thickness of 4 mm, a Clark’s level IV, it was ulcerated and highly melanotic. The bilateral sentinel node biopsy was negative. She entered into a randomized Phase II/III clinical study comparing a vaccine composed of irradiated melanoma cells plus BCG plus GM-CSF versus IFN-alpha 2b and she was assigned to the vaccine arm. During the two years treatment she remained disease-free; the final CAT scan being performed in August 2011. Between November and December 2011, her gynecologist treated her with three cycles of 200 mg progesterone/day for ten days, every two weeks, for ovary dysfunction. In November 2011 the patient returned to the Hospital for clinical and imaging evaluation and no evidence of disease was found. At the next visit in March 2012 an ultrasound revealed multiple, large metastases in the liver. A CAT scan confirmed the presence of liver, adrenal glands and spleen metastases. A needle biopsy of a liver lesion revealed metastatic melanoma of similar characteristics to the original tumor. We suggest that progesterone treatment triggered proliferation of so far dormant micrometastases that were controlled during CSF470 vaccine treatment.

**Conclusion:**

The use of progesterone in patients with melanoma that are under immunological treatments should be carefully considered, since progesterone could modify the balance of pro-inflammatory and Th1 functions to a regulatory and anti-inflammatory profile of the immune system that could have an impact in tumor progression.

## Background

The reports associating melanoma progression and progesterone (Pg) exposure during pregnancy or the use of oral contraceptives have been controversial. Many authors have reported a poor prognosis in pregnant women with melanoma as compared to non-pregnant women’s tumor. Several retrospective reviews showed a worsened prognosis in pregnant women with melanoma and found that PgR and ER can be detected in melanoma tissue [1]. Instead, recent data found no increased risk of CM with the use of exogenous female hormones [2], and the timing of the disease diagnosis during pregnancy did not appear to influence the risk of melanoma mortality [3]. However, significant associations of CM with parity and age of first pregnancy were found, thus warranting further research [2].

Given this scenario, hormonal treatment might affect the immune response to tumor antigens induced in patients who are being vaccinated, but to our knowledge this has not been previously reported.

In this report we present the case of a melanoma patient treated with a melanoma vaccine [4,5] that remained disease-free for two years but afterwards showed a dramatic progression after receiving only one month of Pg therapy for ovary dysfunction.

## Case presentation

**Figure 1:**
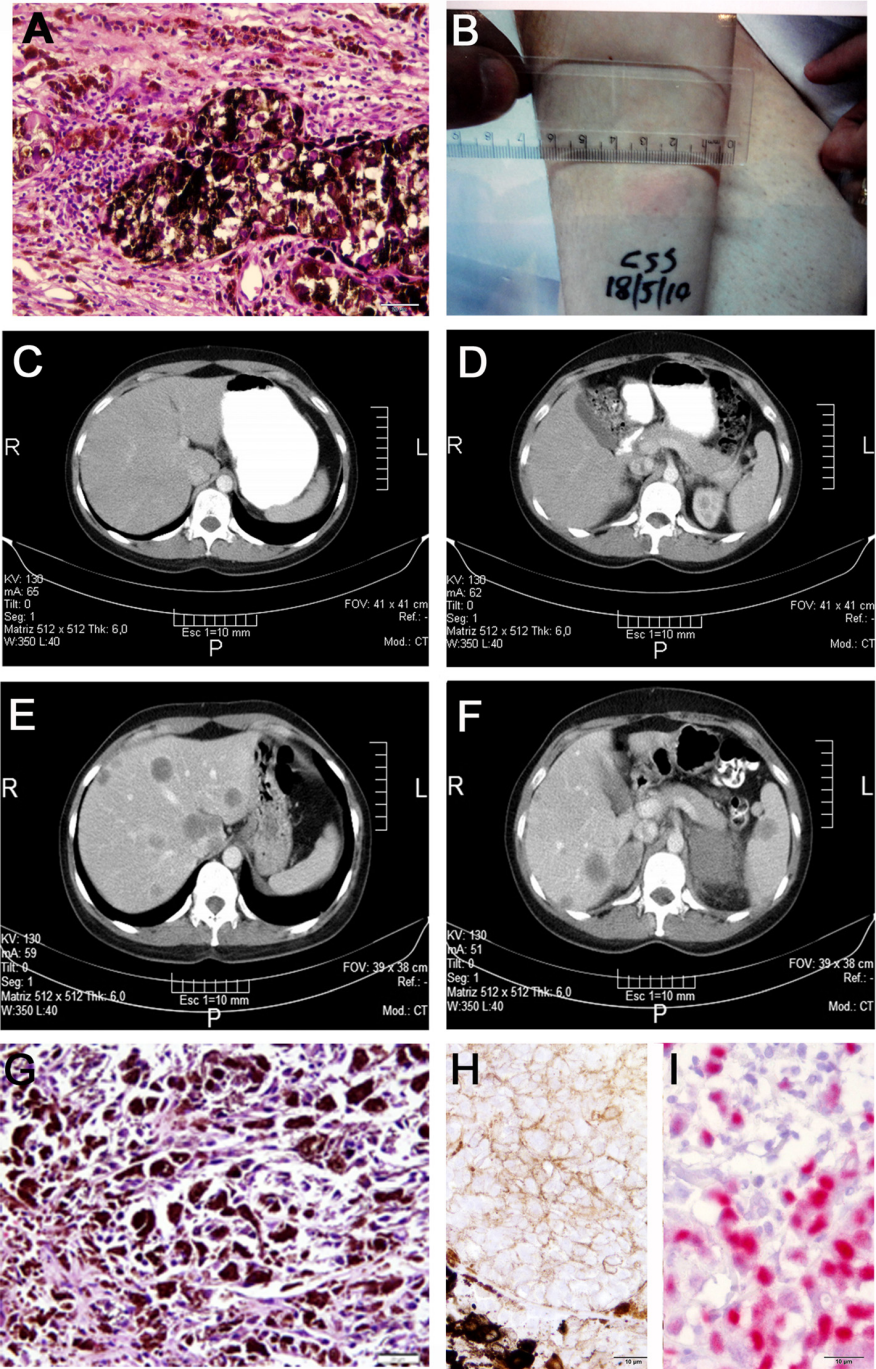
**Pictures of 001-CSS patient melanoma and CAT scans. A**- Micrograph of the primary tumor (hematoxylin/eosin staining) showing highly melanotic tumor cells and scarce lymphocytes in the tumor periphery. Original magnification = 200×; scale bar = 20 μm. **B**- DTH reaction to the vaccine at the right forearm is shown as an example of patient’s immune reaction after receiving eight doses of CSF470 vaccine plus BCG plus GM-CSF, as described in the text. **C **and **D**- CAT scan pictures of the abdomen obtained by the end of the clinical study (August 2011). No melanoma lesions were detected. **E **and **F**- CAT scan pictures obtained at March 2012, three months after the patient received Pg treatment, showing multiple lesions in the liver, suprarenal glands and spleen. **G**- Micrograph of a hepatic needle biopsy (hematoxylin/eosin staining) confirming the presence of multiple melanoma metastases in the hepatic parenchyma. Original magnification = 200×; scale bar = 20 μm. **H**- Immunohistochemical staining for PgR in the primary tumor was negative; **I**- positive control for PgR staining (breast carcinoma). For both, original magnification = 400×; scale bar = 10 μm.

A 33 years-old caucasian woman, nulliparous, presented with a quick, suspicious growth of a congenital nevus located in the intermammary sulcus and which was ulcerated. After surgery in May 2009 she was diagnosed with stage IIC cutaneous epithelioid nodular melanoma, in vertical growth phase, with a Breslow thickness of 4.1 mm and a Clark’s level of IV. The tumor was highly melanotic, ulcerated, and presented scarce lymphocytic infiltration (Figure 1A). The bilateral sentinel lymphnode biopsy was negative. After giving written consent she entered into a randomized Phase II/III clinical study comparing the CSF470 vaccine plus BCG plus GM-CSF versus IFN-alpha, and she was assigned to the vaccine arm. The clinical study (CASVAC0401) has been approved by the Comite Independiente de Etica para Ensayos en Farmacología Clínica, “Profesor Luís M. Zieher” Buenos Aires, Argentina. CSF470 vaccine consists in a mixture of four gamma-irradiated melanoma cell lines injected i.d. plus 10^6^ cfu BCG plus 400 μg rhGM-CSF (divided in four daily injections, 100 μg GM-CSF each). She started the clinical study two months after surgery (July 2009) with a LDH = 287 U/L (normal range 230–460). She received a total of 13 doses of CSF470 vaccine (the first 4 doses, every three weeks, then every two months until completion of the first year and finally every 3 months in the second year) with good tolerance presenting only grade 2 toxicity (erythema, edema and pain) at the vaccination site. DTH reactions were quite strong and lasted at least three days after vaccination (Figure 1B). During the course of the study, a cervical lymph node suspiciously enlarged, but then returned to normality and no biopsy was performed.

In June 2010, a control CAT scan depicted an ovarian cyst that suggested a functional cyst or endometriosis. In August 2011 she completed the clinical study of CSF470 vaccine and she was found to be disease-free by CAT scans of the brain, abdomen, thorax and pelvis (Figure 1C-D). Her serum LDH was 309 U/L, and the patient returned to her hometown.

Between November and December 2011 she was treated by her gynecologist with three cycles of Pg, 200 mg/day for ten days, every two weeks (MAFEL, Raymos Laboratories, Argentina), to treat her ovarian cyst. At that time (November 2011) her serum LDH was 369 U/L and all other blood laboratory values were normal. Chest radiography was normal and abdominal ultrasound only showed a 4 cm diameter ovarian cyst, without adenopaties. No CAT scan was made at that time. In March 19/2012 the patient came to a new follow-up visit and ultrasound showed multiple liver heterogeneous diffuse nodules (20-25 mm) that were further confirmed by CAT scan, along with expansive lesions in both suprarenal glands and a spleen nodule (Figure 1E-F). Her serum LDH value was 767 U/L. A fine needle biopsy of a liver nodule confirmed the presence of melanoma metastasis, with highly melanotic cells (Figure 1G). The patient was treated with two cycles of biochemotherapy [6] plus tamoxifen with no clinical response. Within the next two months the patient developed brain metastases, detected by MNR, and subcutaneous nodules in the neck. BRAF sequencing of the liver biopsy revealed the presence of V600E mutation and the patient started Vemurafenib treatment (960 mg b.i.d) in July 2012, attaining partial remission.

Lack of hormone receptors (ER and PgR) was found in the primary tumor biopsy by immunohistochemistry (Figure 1H and I), but this finding could not be confirmed in the hepatic metastasis biopsy due to lack of material.

## Discussion

It is well known that reproductive steroid hormones, particularly Pg, in addition to its widely recognized effects on endometrial epithelial and stromal cells and spiral arteries, affect the activity of the immune system, inducing active immune tolerance against fetal antigens during pregnancy [7]. The immune-modulatory effects of Pg involve a number of immune effectors, i.e., it blocks mitogen stimulated T cell proliferation [8], it increases secretion of IL-10 by T cell clones [9], it modulates antibody production [10], it decreases the oxidative burst of monocytes [7], and it reduces pro-inflammatory cytokines production by macrophages in response to bacterial products [9]. Pg alters the balance of Th1/Th2 immune profiles towards Th2, inducing IL4, IL5 and IL10 production [9]. Also, IFN-related genes are down-regulated in peripheral blood lymphocytes in women’s luteal phase, when Pg reaches peak levels, as compared to the follicular phase of the cycle [11].

The link between Pg and the immune system is established by lymphocyte Pg receptors expressed in peripheral blood gamma-delta T cells of pregnant women and in peripheral NK cells. Regulation of lymphocyte Pg receptors is dependent on T cell activation, since efficient recognition of fetal antigens is a requirement for the initiation of Pg-dependent immune-regulatory mechanisms [12]. Also, it has been shown that during pregnancy, the severity of diseases caused by inflammatory responses (i.e. multiple sclerosis) is reduced and the severity of diseases that are mitigated by inflammatory responses (i.e. infections like influenza and HPV) is increased [13].

Regulation of host immune system by Pg-based contraceptives treatment of animals and humans can have significant effects on the host immune response. Pg can modulate both the innate and adaptive immune system, resulting in an increased susceptibility of women to viral infections, like HPV or HIV. It has been previously reported that mice treated with depot medroxyprogesterone acetate (DMPA) have decreased levels of HSV-2 specific mucosal immune responses after intravaginal immunization with the attenuated strain of HSV-2 (TK-HSV-2). Consequently, these mice fail to develop protective immune responses against subsequent WT HSV-2 challenge [14]. A more recent study reported that Pg treatment at concentrations achieved during hormone-therapy, decreases the proliferation and Th1-type cytokine production of varicella-zoster virus-specific CD8+ and CD4+ T cells, and this effect was exacerbated in cells obtained from HIV-infected individuals [15]. Since Pg decreases antibody production, CTL activity, IFN-gamma production, and antibody-dependent cell cytotoxicity activity in women using Pg-based contraceptives, these facts may contribute to the increased susceptibility and shedding of HIV-1 observed in women using these hormone therapies [16]. Similarly, Pg could negatively influence specific Th1 and pro-inflammatory immune responses induced by active immunization with tumor vaccines.

In view of the results presented, we hypothesize that as a consequence of CSF470 vaccination, the patient’s melanoma remained under control of the immune system for two years, although her tumor was possibly disseminated as micrometastases below the detection level of CAT scans. Three months after completing Pg treatment for her ovary cysts a rapid disease progression ensued. A possible explanation could be that Pg treatment decreased the activity of immune effectors elicited by the anti-melanoma vaccine and/or increased the function of regulatory T cells, thus unbalancing the patient’s immune system. Since her primary CM showed no PgR expression, a direct mitogenic role of Pg on melanoma cells seems less probable, although we cannot rule out that the metastases could have expressed PgR, since we could not address this point due to scarce material from the liver biopsy.

## Conclusion

We believe that this case brings a word of caution to be considered when cancer immunotherapy is combined or concomitantly administered with hormone therapy, evidencing the delicate interactions established between the immune responses elicited by vaccines, the immune-modulating effect of sex hormones and tumor cells.

## Consent

Written consent for publication of her clinical details and/or clinical images was obtained from the patient. A copy of the consent form is available for review by the Editor of this Journal.

## Abbreviations

ER: Estrogen receptor; Pg: Progesterone; PgR: Progesterone receptor; DMPA: Depot medroxyprogesterone acetate; CAT scans: Computed axial tomography scans; CTL: Cytotoxic T lymphocyte; LDH: Lactate dehydrogenase; IFN-alpha: Alpha Interferon; BCG: Bacillus Calmette-Guerin; Cfu: Colony forming units; GM-CSF: Granulocyte-macrophage colony stimulating factor.

## Competing interests

The authors declare no competing interests.

## Authors’ contributions

JM conceived the study, acquired and interpreted the data and contributed to write the manuscript. IJT helped to draft the manuscript. MMB contributed to review related literature, prepared the figures and helped to write the manuscript. All authors read and approved the final manuscript.

## Authors’ information

JM and MMB are members of the Consejo Nacional de Investigaciones Científicas y Técnicas (CONICET). JM is the Principal Investigator of the CASVAC0401 Clinical Study. IJT is a fellow (PhD student) from CONICET.

## Pre-publication history

The pre-publication history for this paper can be accessed here:

http://www.biomedcentral.com/1471-2407/13/132/prepub
